# DDFC: deep learning approach for deep feature extraction and classification of brain tumors using magnetic resonance imaging in E-healthcare system

**DOI:** 10.1038/s41598-024-56983-6

**Published:** 2024-03-18

**Authors:** Abdus Saboor, Jian Ping Li, Amin Ul Haq, Umer Shehzad, Shakir Khan, Reemiah Muneer Aotaibi, Saad Abdullah Alajlan

**Affiliations:** 1 School of Computer Science and Engineering, University of Electronic Science and Technology of China, Chengdu, 611731 China; 2 Department of Computer Science, Mohi-Ud-Din Islamic University, Azad Jammu Kashmir, Pakistan; 3 College of Computer Science and Information Sciences, Imam Mohammad Ibn Saud Islamic University, Riyadh, 11432 Saudi Arabia; 4 Department of Computer Science and Engineering, University Centre for Research and Development, Chandigarh University, Mohali, 140413 India

**Keywords:** Computational biology and bioinformatics, Diseases

## Abstract

This research explores the use of gated recurrent units (GRUs) for automated brain tumor detection using MRI data. The GRU model captures sequential patterns and considers spatial information within individual MRI images and the temporal evolution of lesion characteristics. The proposed approach improves the accuracy of tumor detection using MRI images. The model’s performance is benchmarked against conventional CNNs and other recurrent architectures. The research addresses interpretability concerns by employing attention mechanisms that highlight salient features contributing to the model’s decisions. The proposed model attention-gated recurrent units (A-GRU) results show promising results, indicating that the proposed model surpasses the state-of-the-art models in terms of accuracy and obtained 99.32% accuracy. Due to the high predictive capability of the proposed model, we recommend it for the effective diagnosis of Brain tumors in the E-healthcare system.

## Introduction

Medical imaging is crucial for accurate diagnosis and early detection of neurological disorders, particularly brain tumors, which can significantly impact morbidity and mortality rates^1^. Traditional methods for brain tumor detection often rely on human expertise, leading to errors. To tackle the traditional brain tumor detection methods problems artificial intelligence (AI) techniques are incorporated to design a computer-aided diagnosis (CAD) system for the detection of brain tumors. The AI-based CAD system extracted more deep features from data for target production. The AI techniques particularly machine learning (ML) and deep learning (DL) techniques have been used by various researchers in designing CAD diagnosis systems for brain tumor detection. However, these methods still have the problem of lack of prediction accuracy according to the literature review. To handle the lack of prediction accuracy problem of early proposed methods of brain a new method is necessary to accurately detect brain tumors at the early stage for proper treatment and recovery. In this research study, we proposed a new AI-based CAD method by incorporating advanced deep learning techniques, particularly gated recurrent units (GRUs), which offer an opportunity to enhance accuracy and efficiency for brain tumor detection. The GRUs, a type of recurrent neural network, excel at capturing sequential patterns in data, making them ideal for modeling complex relationships in medical images^2^. The integration of GRU-based models not only holds the promise of enhancing detection performance but also provides an avenue for understanding the salient features and temporal dynamics associated with brain tumors^3^.

To further improve the predictive performance of GRU model data augmentation and attention mechanisms are integrated to effectively analyze brain tumor MRI imaging data. We commence by curating a diverse collection of image data and applying data augmentation strategies, such as rotations, flips, and color variations, to amplify the range of data variability. Subsequently, formulating a unique GRU-based architecture tailored specifically for image analysis applications. This architectural framework encompasses an encoder module featuring convolutional layers, which are instrumental in extracting meaningful image features. We introduce attention mechanisms, including spatial attention, to empower the model to selectively concentrate on pertinent image regions during the analysis process. The BTD dataset is then utilized for training the GRU model, employing suitable loss functions that align with the specific task, be it classification. The fine-tuning of hyperparameters, encompassing learning rates and batch sizes, optimizes the model’s learning dynamics. Evaluation involves examining how well the model performs on a specific test dataset using appropriate evaluation measures such as accuracy, specificity, sensitivity, f1-score, precision, and AUC. Additionally, deploying the trained model for real-world predictions involves implementing deployment optimization techniques (SGD and ADAM ) like model quantization to enhance operational efficiency. Additionally, the proposed model (A-GRU) performance is compared with state-of-the-art models.

The contributions of this study are summarised as follows:

- AI-based attention-GRU (A-GRU) model is proposed for the precise identification of Brain tumors in the E-healthcare system.
- Attention mechanism integrated with GRU for extraction of more deep features from images to enhance the predictive accuracy.
- Data augmentation techniques are incorporated into enhanced training data to train the model effectively. The model’s assessment is made using original brain MRI imaging data.
- The proposed model (A-GRU) utilizes a variety of metrics to assess outcomes.
- When contrasted with the existing models, the proposed model is performing exceptionally well.

The following is the structure of the remaining portions of the paper: the review of the literature is in “Literature review”. The analysis of the data set and the suggested model methods are covered in detail in “Materials and method”. The experiments are described in detail in “Experiments”. “Conclusion” presents the conclusion and directions for future research.

## Literature review

The brain tumor’s accurate and on-time detection is important for effective treatment and recovery at an early stage in the E-healthcare system. Artificial intelligence (AI) techniques particularly machine learning (ML) and deep learning (DL)-based computer-aided diagnosis (CAD) systems play important roles in the detection of critical diseases. The AI-based CAD system effectively interprets the MRI images of brain tumors as compared to medical doctors. However, AI-based CAD system predictive accuracy of interpreting the images is still not enough to accurately detect the brain tumor. To tackle the accurate detection problem of brain tumors various researchers proposed different methods using machine learning deep learning techniques. In this research work, we have explored various brain tumor detection methods to find the research gap in the domain and designed a new method to accurately detect brain tumors using machine learning and deep learning techniques.

Haq et al.^4^ proposed a new automated diagnostic framework for accurate brain cancer diagnosis, utilizing deep learning techniques in an intelligent integrated model (CNN-LSTM) on BTDS, MBNDS, and BMIDS datasets. The proposed model achieved high accuracy as compared to baseline models. Methil^5^ introduces a novel method using brain imaging to identify brain cancers, utilizing equalization of histograms and methods for opening, followed by a convolutional neural network for classification, achieving high accuracy. In another study, Jia and Chen^6^ proposed a novel approach called fully automatic heterogeneous segmentation using support vector machines (FAHS-SVM) for accurately segmenting brain tumors using deep learning techniques in MRI scans. Manogaran et al.^7^ use a machine learning technique for analyzing orthogonal gamma distributions for brain tumors, identifying abnormalities through the identification of regions of interest. Polly et al.^8^ work presents a computerized system using a support vector machine (SVM) to distinguish between healthy brain MRI images and abnormal brain tumors, achieving high accuracy, sensitivity, and specificity.

Raut et al.^9^ proposed a CNN model for brain tumor detection, trained using pre-processed MRI scans, achieving 95.55% accuracy. K-means clustering is applied to identify specific tumor regions.

In this context, Lu et al.^10^ have recommended a DNN-based framework to address challenges in brain tumor detection using MRS data, including scarcity of training data and potential data corruption. Grampurohit et al.^11^ incorporated deep learning models such as the VGG-16 architecture and a CNN to locate the tumor location on cerebral scan images. In this research, DNN, namely VGG-16 and CNN, was examined with brain MRI imagery, and the proposed model obtained high accuracy. Noreen et al.^12^ proposed a multi-level feature extraction technique for early brain tumor diagnosis using pre-trained deep learning models, achieving high accuracy and superior performance.

Saleh et al.^13^ aim to improve MRI technology accuracy and effectiveness in classifying brain cancers using AI, DL, and CNN techniques. Five models, ResNet50, VGG16, MobileNet, InceptionV3, and Xception, have high precision for unidentified images. In another study, Kumar Mallick et al.^14^ introduce a novel image compression technique called Deep Wavelet Autoencoder (DWA), which combines feature reduction and wavelet transform image decomposition. Using brain images, it achieved 96% accuracy, surpassing existing methods. Irsheidat and Duwairi^15^ present a CNN model that accurately predicts brain tumors from MRI scans, achieving a predictive accuracy of up to 88.25% on test data and 96.7% on evaluation data.

Table 1 presents a comprehensive overview of the literature, aiming to enhance comprehension of the research gap within the previously suggested models. The reviewed and investigated literature demonstrates that there is still a deficiency in the prediction accuracy of the current methods for diagnosing brain tumors to appropriately treat and recover patients in the E-healthcare system. For these reasons, the E-healthcare systems medical experts have not been using the AI-based CAD diagnosis tools that are currently available to diagnose diseases like brain tumors. An innovative, reliable CAD diagnosis system based on AI approaches is required to address this problem in order to properly diagnose and treat brain tumors in an E-healthcare system.

**Table 1:** Summary of brain tumor diagnosis models.

References	Feature extraction algorithm	Dataset	Classification models	No. of classes	Metrics
^1^	VGG-19 CNN	BTD, Radiopaedia	VGG-19 CNN	Three classes	Sensitivity 88.41%, specificity 96.12%, accuracy 94.51%
^4^	CNN, LSTM	BTDS, MBNDS, BMIDS	CNN-LSTM	Three classes	99.22% accuracy
^5^	Histogram equalization, erosion, dilation	BTD	CNN	Two classes	Training recall 98.55%, validation recall 99.73%
^16^	CNN	BTDS	ResNet-CNN	Three classes	99.90% accuracy, 100% specificity, 89.60% sensitivity
^6^	ELMA	BTD	FAHS-SVM	Two classes	98.51% accuracy
^17^	–	MRI and SPECT images	KNN, SVM	Two classes	96.8% accuracy, 95%, precision, 91%, f1-score
^8^	PCA, DWT	MRI images	SVM	Two classes	99% accuracy
^18^	CNN	MRI images	Resnet50, InceptionV3 and MobileNetV2	Three classes	98.47%, 95.41%, 92.36% accuracy of models Resnet50, MobileNetV2 and InceptionV3
^19^	DNN	MRI images	H-DNN	Two classes	98.7% accuracy
^20^	Xception and IRV2	BraTS 2018 dataset	Two-channel DNN	Two classes	93.69% accuracy
^11^	VGG-16, CNN	MRI images	CNN, VGG-16	Two classes	CNN 93.36%, VGG-16 97.16%
^21^	Inception-v3, DenseNet201	BD	Xception-Ensemble	Three classes	Inception-v3 94.34% accuracy , DenseNet201 94.34% accuracy
^13^	Xception	MRI images	Xception, ResNet50, InceptionV3, VGG16, MobileNet	Three classes	Xception, ResNet50, InceptionV3, VGG16, and MobileNe, obtained F1-score, 98.75%, 98.50%, 98.00%, 97.50%, and 97.25% respectively
^14^	DWA	RIDER	AE-DNN	Two classes	96% accuracy
^15^	ACNN	MRI images	ACNN	Two classes	96.7% accuracy

## Materials and method

### BTD dataset

**Figure 1:**
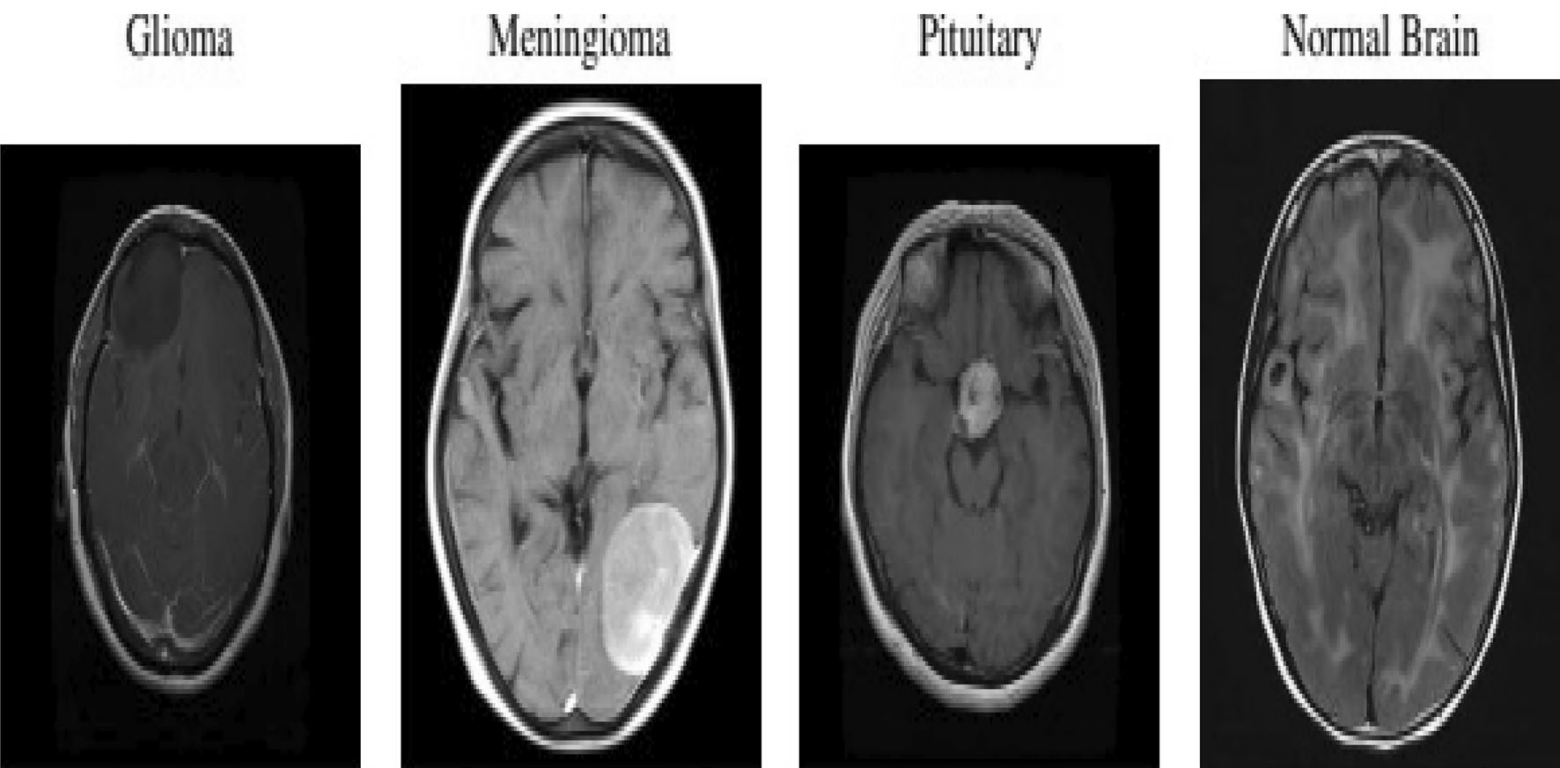
Standardized MRI images of various tumor types and orientations.

The utilized dataset of image-based content consisted of 1311 magnetic resonance images (MRI)^22^ images enhanced with T1-weighted contrast from Kaggle. This dataset encompassed four distinct image categories: glioma (300 images), meningioma (306 images), pituitary gland tumor (300 images), and normal brain scans (405 images). All images were oriented in sagittal, axial, and coronal planes. Examples of the diverse tumor types and their respective planes are illustrated in Fig. 1. It’s worth noting that the image count varied for each patient. The MRI within this dataset exhibited varying dimensions. These images served as input for the networks and were consequently resized to dimensions of 80 by 80 pixels. Each image underwent two transformations for dataset augmentation. The initial alteration encompassed a 90^∘^ rotation, while the second involved vertical image flipping^23^.

### Gated recurrent unit (GRU)

**Figure 2:**
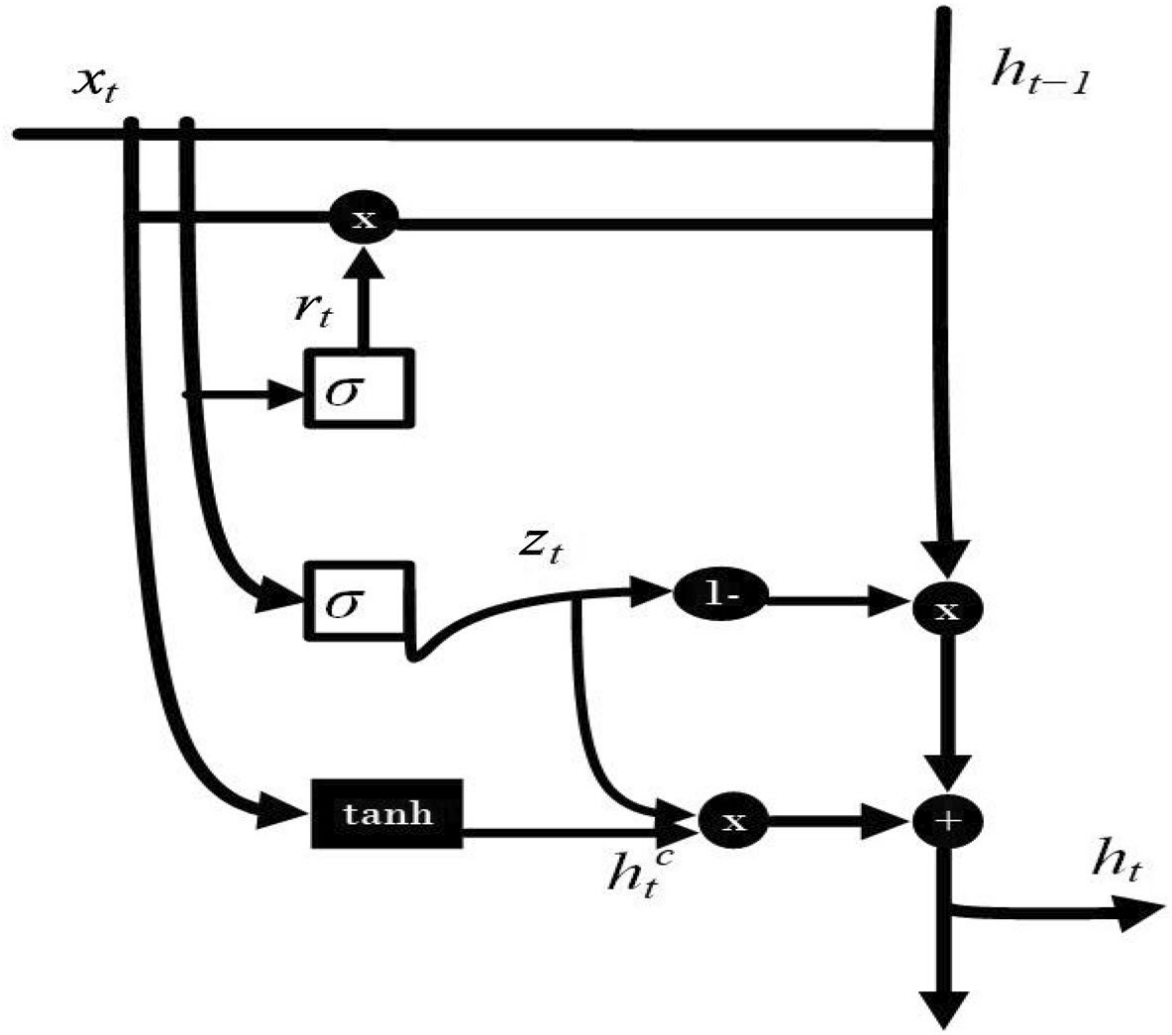
The architecture of the GRU model.

The gated recurrent unit (GRU) is an architecture within the realm of recurrent neural networks (RNNs)^24^ that has been devised for the purpose of handling and modeling sequential data. It addresses certain limitations that are encountered in conventional RNNs, particularly the issues of vanishing gradients and the complexities associated with capturing long-range dependencies within sequences. It was also presented as a modification of the conventional long short-term memory (LSTM)^25^ model. Both the GRU and LSTM architectures are formulated to tackle the challenge of the vanishing gradient problem that can occur during the training of deep networks involving sequential data, like text or time series. But GRU is a more straightforward design featuring just two gates: reset and update. This leads to quicker training and inference processes as a result of having fewer parameters. This design works particularly well for smaller datasets and tasks that involve simpler relationships. However, it might not be as effective in capturing complex, long-range dependencies. The distinctive feature of GRUs lies in their incorporation of gating mechanisms, which serve as controls governing the flow of information throughout the network. The GRU model structure is given in Fig. 2.

Here’s a breakdown of the primary components of a GRU. GRU has four components: update gate, reset gate, candidate hidden state, and final hidden state. Equations (1), (2), (3), and (4) define the computational processes carried out by the GRU.

1 $$\begin{aligned} r_t= & {} \sigma (W_{xr}x_t + W_{hr}h_t-1 + b_r) \end{aligned}$$

2 $$\begin{aligned} z_t= & {} \sigma (W_{xz}x_t + W_{hz}h_t-1 + b_z) \end{aligned}$$

3 $$\begin{aligned} h_t^c= & {} \tanh \left( W_{xc}x_t + r_t \odot (W_{hc}h_{t-1}) + b_c\right) \end{aligned}$$

4 $$\begin{aligned} h_t= & {} (1 - z_t) \odot h_{t-1} + z_t \odot h_{tc} \end{aligned}$$

In the preceding computation, where $$x_t$$ signifies the input at time step *t*, $$ h_t-1$$ represents the previous hidden state at time step $$t-1$$. The symbol $$\sigma $$ denotes the sigmoid activation function, while $$W_{xr}$$ and $$W_{hr}$$ stand for the weight matrices pertaining to the input-to-reset-gate and hidden-to-reset-gate connections respectively. The term $$b_r$$ corresponds to the bias component. The symbol $$\odot $$ denotes element-wise multiplication. Similarly, $$W_{xc}$$ and $$W_{hc}$$ represent the weight matrices associated with the input-to-candidate-hidden-state and reset-gated-hidden-to-candidate-hidden-state connections respectively. The term $$b_c$$ stands for the bias, and the activation function *tanh* is the hyperbolic tangent^26^.

### Attention mechanism

The reduction in prediction accuracy for the output sequence within the GRU network is observed when employing a more extensive input sequence. This occurs because the network treats all input variables uniformly, overlooking potential variations in their correlations with the forecasting task. To mitigate this issue, an attention mechanism can be implemented to highlight more pertinent input variables.

The attention mechanism is composed in the form of an attention vector-generating encoder based on the encoder output as input for a decoder that creates a hidden state based on the input. Each step of hidden state is given an attention score by the encoder, which uses the hidden state of the previous view decoder to divide the hidden states. After that, a soft-max operation is used to the attention scores to create an attention vector. Consequently, during the prediction of the output value by the decoder, the encoder directs attention towards input variables that are similar to the predicted value.

The attention mechanism functions by assigning weights or conferring significance to each lower level during the assessment of the higher-level representation, ultimately concentrating on a pivotal segment of the textual content. Equations (5), (6), and (7) describe the attention mechanism mathematically^27^.

5 $$\begin{aligned} M= & {} tanh(H) \end{aligned}$$

6 $$\begin{aligned} \alpha= & {} \text {softmax}(w^TM) \end{aligned}$$

7 $$\begin{aligned} C= & {} H \alpha ^T \end{aligned}$$

Here, H symbolizes a matrix comprising hidden output vectors (h1, h2,...hN) *N* specifies the count of hidden layers generated by GRU, *C* denotes the attention model’s contextual word, *w* represents the trained vector, and $w^T$ stands for its transpose. The attention weights ${( \alpha 1, \alpha 2,\ldots \alpha N )}$ are employed to create the vectors *C* as well as the output sentence representation $h^*$ for a provided input, as shown in Eq. (8).

8 $$\begin{aligned} h^* = \tanh (c) \end{aligned}$$

In the specific context of tumor detection using GRU, this mechanism is likely instrumental in pinpointing crucial attributes that contribute to precise tumor detection. In the area of imaging for healthcare, attention can operate in various ways: spatially, by emphasizing areas potentially containing tumors; temporally, by capturing significant moments in sequences; or across diverse data modalities, by enhancing the model’s decision-making process. This is achieved by assigning differing levels of importance to distinct data elements, allowing the model to prioritize informative regions while disregarding noise. Therefore, this method improves the capability of the model to detect intricate details and elevate diagnostic precision. The specific application of this mechanism hinges on the objectives of the study, the attributes of the information at hand, and the design of the model itself.

### Data augmentation

The data augmentation technique is frequently employed in machine learning^28^, especially within the realm of computer vision. Its purpose is to expand a training dataset synthetically by implementing different changes to the initial data. Concerning images, data augmentation encompasses a spectrum of alterations like rotations, flips, and translations, as well as adjustments in color, brightness, and contrast. Augmenting data primarily aims to increase a model’s capability to generalize effectively and achieve favorable performance on new, unfamiliar data by acquainting it with a broader array of variations. The most commonly used method for data augmentation combines color enhancement with geometric image alterations. In the context of alterations, the lowing operations are typically defined as rotation, reflection, scaling (zooming in/out), and shearing. To increase the quantity of training samples for deep neural networks, distortions of geometry or deformations are commonly used. This serves the purpose of both enhancing dataset size and improving the efficiency of the models. These distortions are often realized through affine transformations, yet they continue to be a subject of ongoing research^29^.

### Cross validation approach

For model validation, we employed the holdout cross-validation technique. In this approach, 30% of each brain tumor MRI dataset was set aside for testing, while the remaining 70% was used for training. Hold-out cross-validation entails splitting the dataset into two parts, a training set and a different testing set. The model is evaluated on the testing set of data after it has been trained on the training set. This approach is relatively straightforward but is particularly valuable when working with large datasets, allowing for the allocation of a portion for testing^30,31^. Usually, the training set contains a more significant proportion of the data, while the testing set is reserved for assessing the model’s capacity to generalize.

### Performance evaluation metrics

To ascertain the efficacy and quality of machine learning models, performance evaluation is essential. The selection of appropriate metrics depends on the particular problem, whether it’s classification, regression, or clustering, and the characteristics of the data. Here, we present a set of frequently used performance evaluation metrics specifically tailored for classification tasks^32^.

Performance evaluation metrics in classification are employed to gauge the effectiveness of machine learning models, particularly in tasks such as binary or multiclass classification. These metrics play a crucial role in quantifying a model’s capacity to make accurate predictions and identifying its areas of proficiency and limitations^33–35^.

Below, there is a selection of widely used classification performance evaluation metrics, complete with their mathematical formulations in Eqs. (9), (10), (11), (12), and (13).

9 $$\begin{aligned} Accuracy= & {} \frac{(TP+TN)}{(TP+TN+FP+FN)} \times 100 \end{aligned}$$

10 $$\begin{aligned} Recall= & {} \frac{TP}{(TP+FN)} \times 100 \end{aligned}$$

11 $$\begin{aligned} Specificity= & {} \frac{TN}{(TN+FP)} \times 100 \end{aligned}$$

12 $$\begin{aligned} Precision= & {} \frac{TP}{(TP+FP)} \times 100 \end{aligned}$$

13 $$\begin{aligned} F1-s= & {} 2\times \frac{Presion \times Recall}{(Precision + Recall)} \times 100 \end{aligned}$$

(where: true negatives (TN), false negatives (FN), true positives (TP), and false positives (FP) are the acronyms for these concepts.)

To calculate the area under the curve (AUC), the receiver operating characteristic (ROC) curve is plotted against the false positive rate at different categorization thresholds, representing the true positive rate (sensitivity). While there isn’t a straightforward mathematical equation to directly compute the AUC, it can be estimated through numerical integration methods. Typically, the AUC is computed using software libraries or tools like Python’s sci-kit-learn, which offer functions to calculate it based on the ROC curve^36^.

### Proposed methodology using GRU with data augmentation and attention

The proposed methodology harnesses the power of GRU (gated recurrent unit) in conjunction with data augmentation and attention mechanisms to enhance the efficacy of analyzing imaging data. We commence by curating a diverse collection of image data and applying data augmentation strategies, such as rotations, flips, and color variations, to amplify the range of data variability. We are subsequently, formulating a unique GRU-based architecture tailored specifically for image analysis applications. This architectural framework encompasses an encoder module featuring convolutional layers, which are instrumental in extracting meaningful image features. We introduce attention mechanisms, including spatial attention, to empower the model to concentrate on pertinent image regions during the analysis process selectively. The BTD dataset is then utilized for training the A-GRU model, employing suitable loss functions that align with the specific task, be it classification. The fine-tuning of hyper-parameters, encompassing learning rates and batch sizes, optimizes the model’s learning dynamics.

Evaluation involves examining how well the model performs on a specific test dataset using appropriate evaluation measures such as mean squared error or accuracy. Additionally, deploying the trained model for real-world predictions involves implementing deployment optimization techniques like model quantization to enhance operational efficiency. To ensure the model’s consistency and precision in real-world scenarios, the proposed model establishes continuous monitoring within a production environment. Regular model retraining with a fresh dataset serves to continually refine the model’s performance and adaptability. Remaining tuned to the latest research breakthroughs in GRU architectures, attention mechanisms, and data augmentation strategies for imaging data further enables us to explore innovative avenues for advancing our proposed methodology.

**Figure 3:**
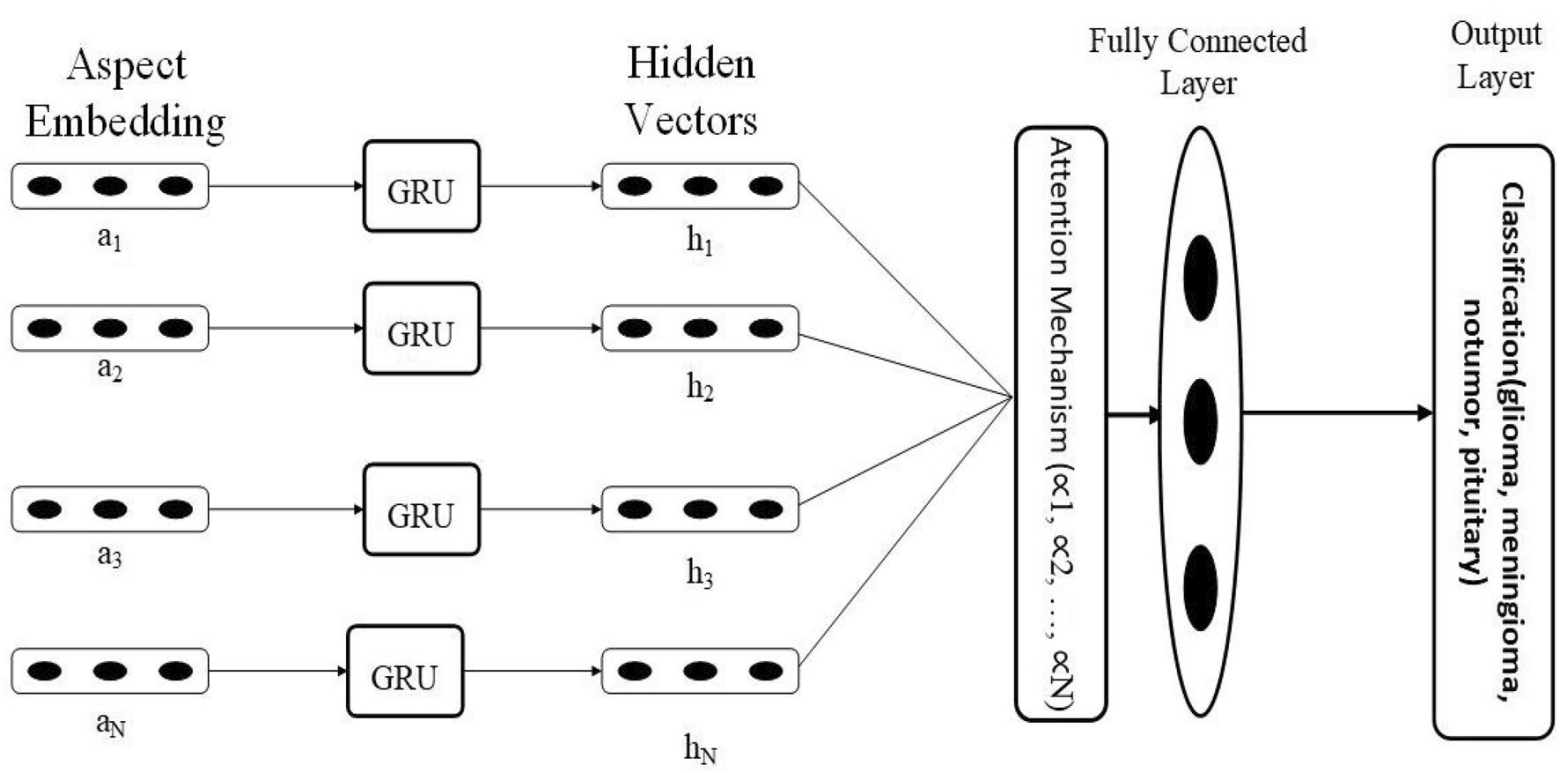
The proposed attention-GRU model (A-GRU) for brain tumor detection.

Considering the domain of brain tumor detection, our methodology takes on heightened relevance. Within the realm of medical diagnostics, the integration of our approach with imaging data holds the potential to elevate the precision of brain tumor identification through the synergistic utilization of attention-GRU with data augmentation, and attention mechanism. This endeavor seeks to foster enhanced early detection, consequently bolstering patient prognosis. By maintaining a trajectory of ongoing improvement and alignment with contemporary research trajectories, our methodology stands poised to offer a promising avenue for pushing the boundaries of medical imaging and healthcare diagnostic practices. Algorithm 1 provides the proposed model pseudocode, whereas Fig. 3 displays the flow chart of the model.

**Algorithm 1:**
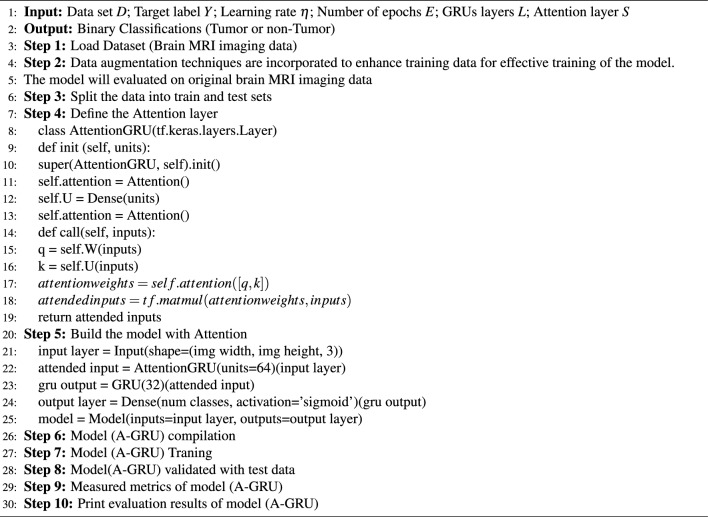
Attention-GRU (A-GRU) model for brain tumor detection.

## Experiments

### Experimental setup

To evaluate the performance of the proposed model (A-GRU) different experiments have been conducted. The brain tumor images data set has been used for the training and validation of the model. The hold-out validation technique has been incorporated and data is divided into 70% and 30% for training and testing of the model. For training parameter optimization, we configured the model with 140 epochs using optimizer ADAM and SGD^37^, with a batch size of 120 and a mini-batch size of 9. Furthermore, we set $\alpha$, the learning rate (LR), to 0.0001. Relu is the inner activation function of the model in all tests, whereas sigmoid is used as the outer activation function. Also, different performance evaluation metrics including accuracy, specificity, sensitivity, precision, F1-score, and the area under the receiver operating characteristic (ROC) curve are computed for model evaluation.

A Windows 8 installed on a computer with a CPU and GPU was the hardware configuration for every experiment that was carried out. All of the experiment software requires Python v3.7. TensorFlow v1.12 will be the back-end software, and Keras v2.2.4 will be used as a high-level API to implement the CNN, LSTM, BiLSTM, GRU and Attention+GRU models. We have iterated three to five times in each experiment to acquire stable results, ensuring consistent and dependable results.

### Results and analysis

#### Data gathering and preprocessing procedures

To validate our model, we used BTD datasets, which included contrast-enhanced T1-weighted images from 233 patients classified into three different kinds of brain tumors: meningioma, glioma, and pituitary. A grand total of 3064 image slices in total were obtained. There were 91 cases with 1426 image slices in the Glioma group and 82 subjects with 708 image slices in the Meningioma category. Simultaneously, the pituitary class encompassed 60 subjects, contributing 930 slices. The class distribution revealed an imbalance among the various groups, potentially leading to overfitting. To address this imbalance and ensure fair representation of cases of pituitary, meningioma, and glioma in the record, we implemented data augmentation techniques. These techniques involved transformations of the original samples through rotation, zooming, and adjustments to brightness. Consequently, the dataset was rebalanced. This augmentation process led to the creation of additional data to effectively train our model. It involves moving images at a 45^∘^ angle from right to left while rotating them along the x-axis, as well as enhancing zooming and brightness for all image types within the original dataset. As a result of these augmentation procedures, the newly generated dataset expanded to a size of 21,448 samples.

####  Results of CNN on original data set

The CNN model’s results were assessed. The hyperparameter configuration involved the application of SGD and ADAM optimizers (OP) with a 0.001 learning rate (LR). We utilized two distinct optimization strategies to compare the outcomes of the proposed A-GRU methodology. With a constant batch size of 120, the model was trained across 140 epochs. To evaluate the model’s performance, various metrics, including accuracy (Ac), specificity (Sp), sensitivity (Sn), precision (Pr), AUC, or area under the curve, and F1-score (F1-S), were evaluated.

**Figure 4:**
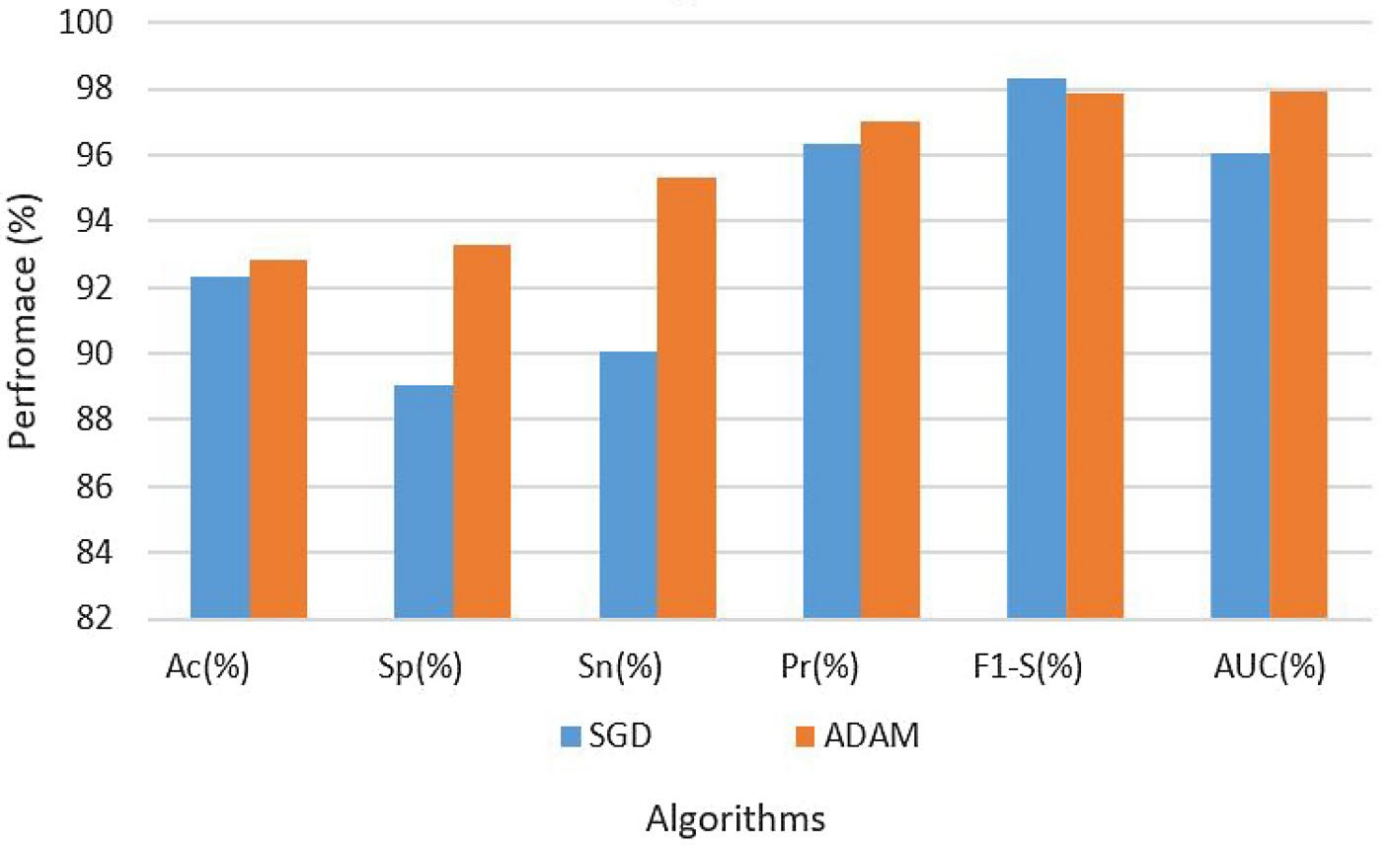
Results of CNN on original dataset.

Table 2 gives a thorough rundown of the experimental findings and hyperparameters. According to the table, the CNN architecture on the original dataset using LR of 0.001 with the SGD optimization technique obtained a 92.33 accuracy rate, specificity of 89.02%, a level of sensitivity of 90.08%, a precision of 96.34%, F1 score of 98.30%, and AUC of 96.05%. On the other hand, the CNN architecture on the original dataset with the ADAM optimization algorithm and the same LR of 0.001 produced a resultant accuracy of 92.84%, specificity of 93.30%, sensitivity of 95.34%, precision of 97.00%, F1 score of 97.89%, and AUC of 97.93%. The findings are also depicted in Fig. 4.

**Table 2:** Results of CNN on original dataset.

Data set	Parameters		Assessment metrics					
	Optimizer	LR	Acc (%)	Sp (%)	Sn (%)	Pre (%)	F1-S (%)	AUC (%)
Original	SGD	0.0001	92.33	89.02	90.08	96.34	98.30	96.05
Original	ADAM	–	92.84	93.30	95.34	97.00	97.89	97.93

####  Results of CNN on augmented data set

**Figure 5:**
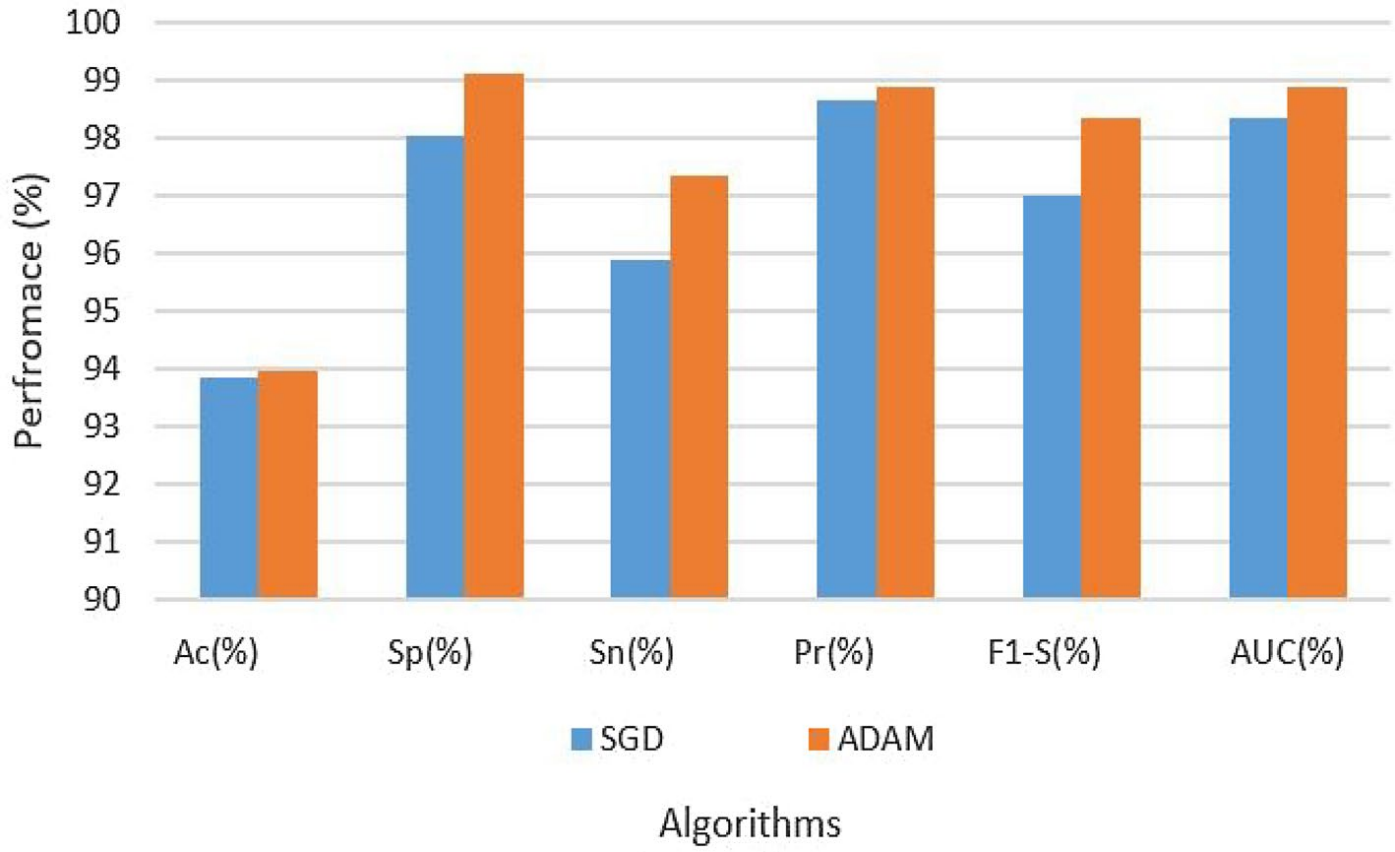
Results of CNN on augmented dataset.

Table 3 provides an exhaustive overview of the hyperparameters and experimental results. According to the table, with an LR of 0.001 and the SGD optimization procedure, with the augmented data set, the CNN’s architectural design was able to achieve 93.87% accuracy, 98.03% specificity, 95.90% sensitivity, 98.64% precision, 97.00% F1 score, and 98.34% AUC. Conversely, the CNN architecture on an enhanced dataset using the ADAM optimization technique with the same LR of 0.001 produced results with 93.98% accuracy, 99.10% specificity, 97.34% sensitivity, 98.89% precision, 98.33% F1 score, and 98.90% AUC. The findings are also depicted in Fig. 5.

**Table 3:** Results of CNN on augmented dataset.

Data set	Parameters		Assessment metrics					
	Optimizer	LR	Acc (%)	Sp (%)	Sn (%)	Pre (%)	F1-S (%)	AUC (%)
Augmented	SGD	0.0001	93.87	98.03	95.90	98.64	97.00	98.34
Augmented	ADAM	–	93.98	99.10	97.34	98.89	98.33	98.90

####  Results of attention-CNN on augmented data set

**Figure 6:**
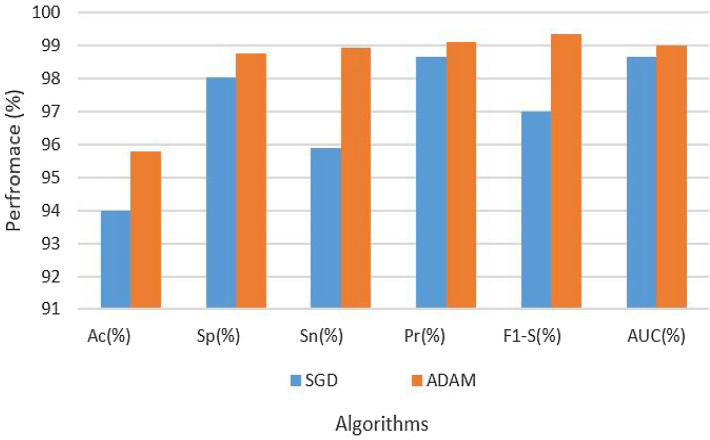
Results of attention-CNN on augmented dataset.

Table 4 provides a comprehensive overview of the hyperparameters and experimental results. In the table, two different optimization algorithms, SGD and ADAM, were used with a fixed learning rate of 0.0001 to assess the performance of Attention-CNN with augmented dataset. The results show that the ADAM optimizer outperforms SGD across most evaluation metrics. The approach specifically obtained 95.78% accuracy, an excellent specificity of 98.77%, exceptional sensitivity of 98.94%, precision of 99.09%, a remarkable F1-Score of 99.34%, and an AUC of 99.00% when it was used with the ADAM optimizer. On the other hand, the SGD optimizer yielded somewhat inferior outcomes, with 94.00% accuracy, 98.03% specificity, 95.90% sensitivity, 98.64% precision, 97.00% F1-Score, and 98.64% AUC. These findings underscore the superiority of the ADAM optimizer in achieving better overall model performance. The results are also shown in Fig. 6.

**Table 4:** Results of attention-CNN on augmented dataset.

Data set	Parameters		Assessment metrics					
	Optimizer	LR	Acc (%)	Sp (%)	Sn (%)	Pre (%)	F1-S (%)	AUC (%)
Augmented	SGD	0.0001	94.00	98.03	95.90	98.64	97.00	98.64
Augmented	ADAM	–	95.78	98.77	98.94	99.09	99.34	99.00

#### Results of the LSTM on original data set

**Figure 7:**
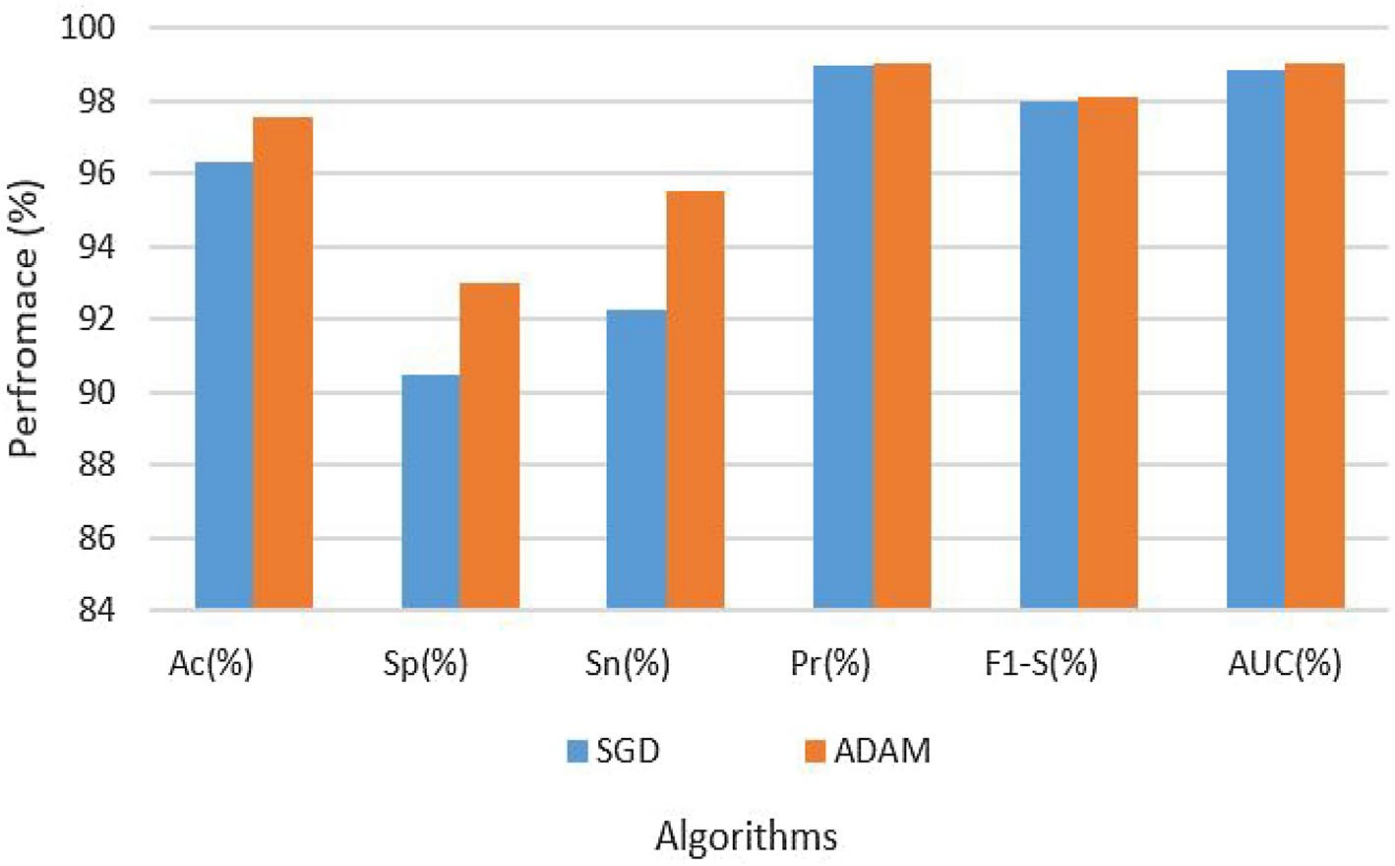
Results of LSTM on original dataset.

Table 5 provides a comprehensive overview of the hyperparameters and experimental results. The table presents the results of the LSTM model on the original dataset using two different optimization algorithms, SGD and ADAM, both with a fixed learning rate of 0.0001. Notably, in most of these criteria, the ADAM optimizer performs better than SGD. The model obtained remarkable results with the ADAM optimizer: 97.54% accuracy, 93.00% specificity, 95.49% sensitivity, 99.00% precision, 98.08% F1-Score, and 99.00% AUC. With an accuracy of 96.30%, a specificity of 90.50%, a sensitivity of 92.28%, a precision of 98.98%, an F1-Score of 98.00%, and an AUC of 98.84%, in comparison, the SGD optimizer produced somewhat lower performance measures. These findings underscore the superior performance of the ADAM optimizer in achieving better overall model results. Figure 7 also presents the results.

**Table 5:** Results of LSM on original dataset.

Data set	Parameters		Assessment metrics					
	Optimizer	LR	Acc (%)	Sp (%)	Sn (%)	Pre (%)	F1-S (%)	AUC (%)
Original	SGD	0.0001	96.30	90.50	92.28	98.98	98.00	98.84
Original	ADAM	–	97.54	93.00	95.49	99.00	98.08	99.00

#### Results of the LSTM on augmented data set

**Figure 8:**
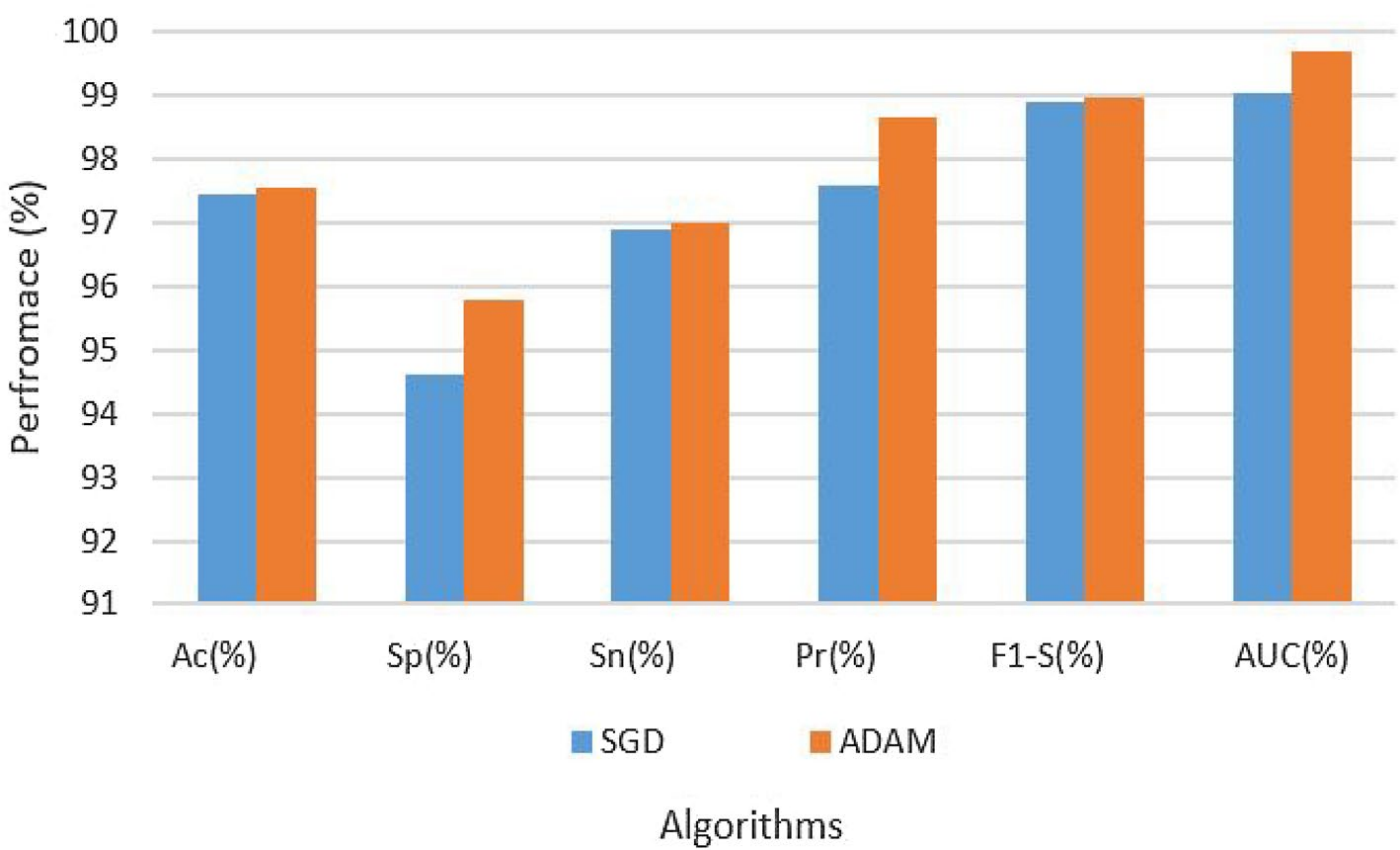
Results of LSTM on augmented dataset.

Table 6 provides a comprehensive overview of the hyperparameters and experimental results. According to the table, the LSTM architecture on the augmented dataset utilizing an LR of 0.001 and the SGD optimization approach, achieved an accuracy of 97.44%, specificity of 94.60%, sensitivity of 96.88%, precision of 97.58%, F1 score of 98.90%, and AUC of 99.04%. On the other hand, the LSTM architecture on an augmented dataset with the ADAM optimization algorithm and the same LR of 0.001, 97.54% accuracy, 95.77% specificity, 97.01% sensitivity, 98.66% precision, 98.98% F1 score, and 99.70% AUC were obtained. The findings are also depicted in Fig. 8.

**Table 6:** Results of LSM on augmented dataset.

Data set	Parameters		Assessment metrics					
	Optimizer	LR	Acc (%)	Sp (%)	Sn (%)	Pre (%)	F1-S (%)	AUC (%)
Augmented	SGD	0.0001	97.44	94.60	96.88	97.58	98.90	90.04
Augmented	ADAM	–	97.54	95.77	97.01	98.66	98.98	99.70

####  Results of the attention-LSTM on augmented data set

**Figure 9:**
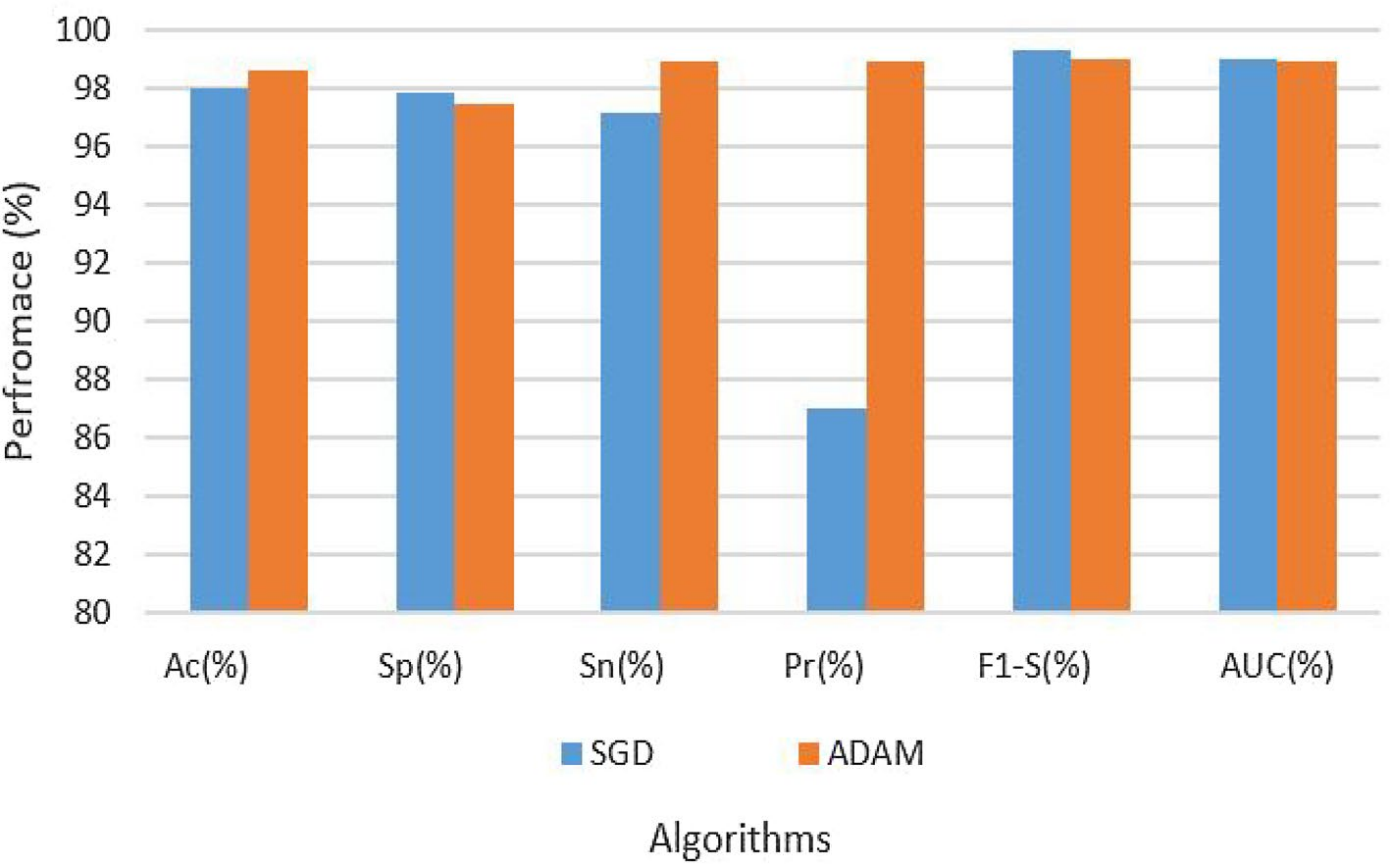
Results of attention-LSTM on augmented dataset.

Table 7 provides a comprehensive overview of the hyperparameters and experimental results. The table displays the performance metrics of attention-LSTM architecture on the augmented dataset, with two different optimization algorithms, SGD and ADAM, both utilizing an LR of 0.0001. Notably, the ADAM optimizer consistently outperforms SGD across most of these metrics. The model demonstrated remarkable results with the ADAM optimizer: 98.64% accuracy, 97.47% specificity, 98.91% sensitivity, 98.96% precision, 98.98% F1-Score, and 98.90% AUC. On the other hand, the SGD optimizer generated comparatively poor outcomes, with 98.00% accuracy, 97.82% specificity, 97.18% sensitivity, 87.00% precision, 99.30% F1-Score, and 99.04% AUC. These findings highlight the superior performance of the ADAM optimizer in achieving better overall model results. The results are also illustrated in Fig. 9.

**Table 7:** Results of attention-LSM on augmented dataset.

Data set	Parameters		Assessment metrics					
	Optimizer	LR	Acc (%)	Sp (%)	Sn (%)	Pre (%)	F1-S (%)	AUC (%)
Augmented	SGD	0.0001	98.00	97.82	97.18	87.00	99.30	99.04
Augmented	ADAM	–	98.64	97.47	98.91	98.96	98.98	98.90

####  Results of the GRU on original data set

**Figure 10:**
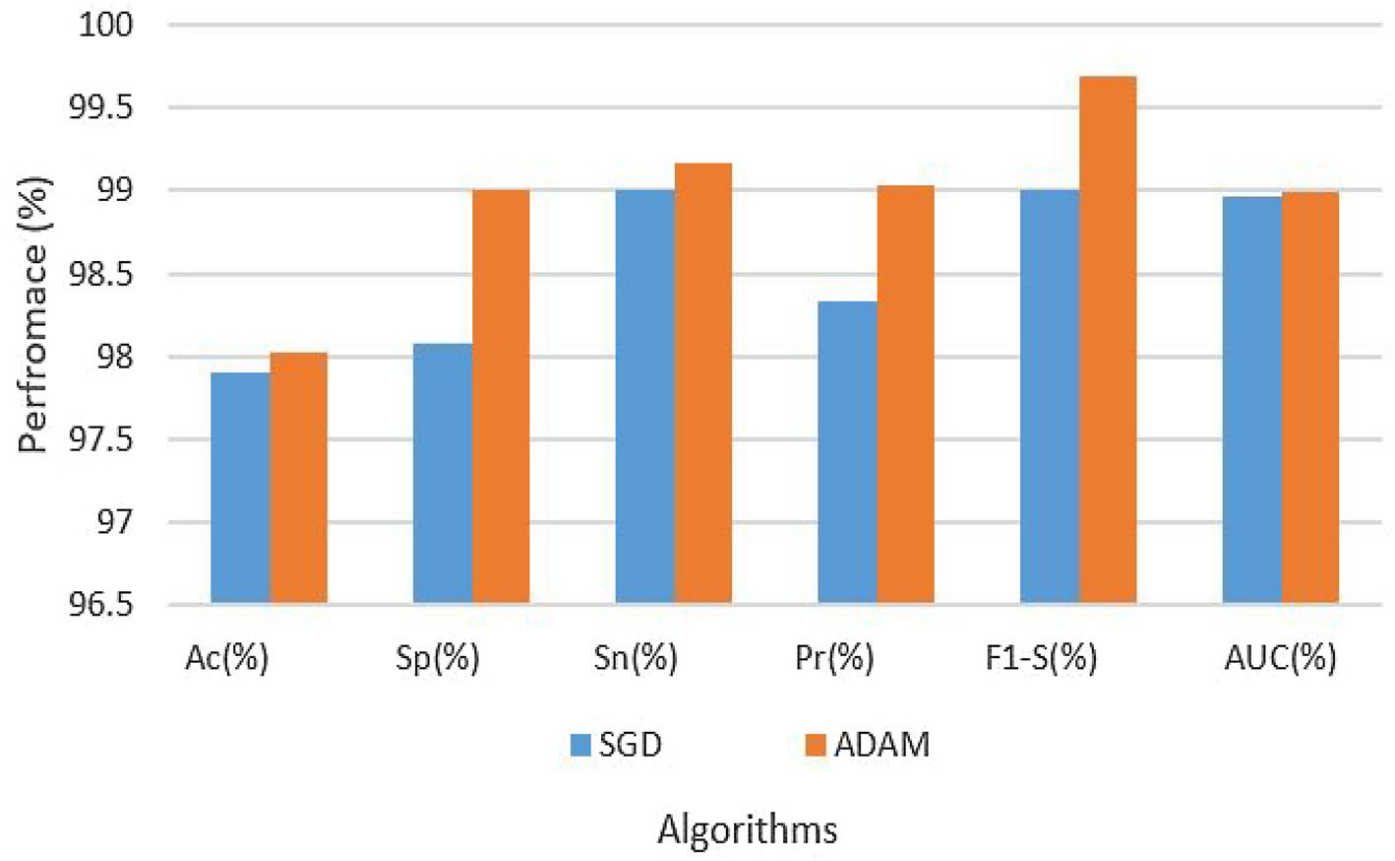
Results of GRU on original dataset.

Table 8 provides a comprehensive overview of the hyperparameters and experimental results. The GRU model’s outcomes on the original dataset are compiled in the table, with two different optimization algorithms, SGD and ADAM, using a constant learning rate of 0.0001. Notably, the ADAM optimizer consistently outperforms SGD across most of these metrics. When utilizing the ADAM optimizer, the model achieved a high accuracy of 98.02%, an exceptional specificity of 99.00%, a strong sensitivity of 99.17%, a precision of 99.03%, an impressive F1-Score of 99.69%, and an AUC of 98.99%. In contrast, the SGD optimizer yielded slightly lower results, with an accuracy of 97.90%, a specificity of 98.09%, a sensitivity of 99.00%, a precision of 98.34%, an F1-Score of 99.00%, and an AUC of 98.97%. These findings underscore the superior performance of the ADAM optimizer in achieving better overall model results. The findings are also presented in Fig. 10.

**Table 8:** Results of GRU on original dataset.

Data set	Parameters		Assessment metrics					
	Optimizer	LR	Acc (%)	Sp (%)	Sn (%)	Pre (%)	F1-S (%)	AUC (%)
Original	SGD	0.0001	97.90	98.09	99.00	98.34	99.00	98.97
Original	ADAM	–	98.02	99.00	99.17	99.03	99.69	98.99

####  Results of the GRU on augmented data set

**Figure 11:**
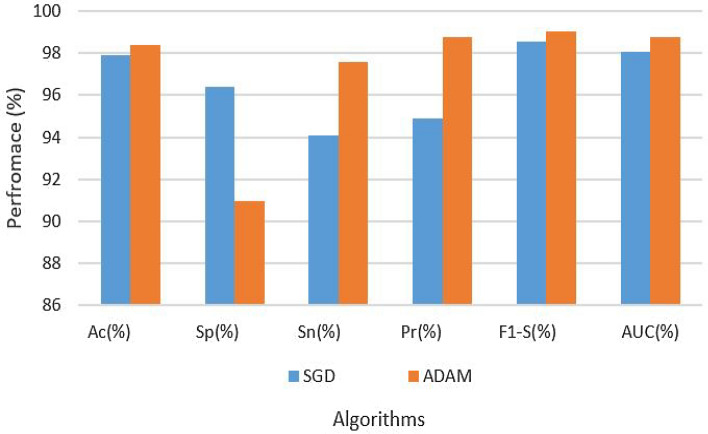
Results of GRU on augmented dataset.

Table 9 provides a comprehensive overview of the hyperparameters and experimental results. As per the table, the GRU architecture achieved an accuracy of 97.92%, specificity of 96.39%, sensitivity of 94.09%, precision of 94.87%, F1 score of 98.56%, and AUC of 98.06% on the augmented dataset using the SGD procedure for optimization and a 0.001 learning rate. In contrast, the LSTM architecture, also on the augmented dataset, utilizing the ADAM optimization algorithm with the same LR of 0.001, attained a 98.40% accuracy, 90.98% specificity, 97.56% sensitivity, 98.78% precision, 99.01% F1 score, and 98.79% overall. These results are also visually represented in Fig. 11.

**Table 9:** Results of GRU on augmented dataset.

Data set	Parameters		Assessment metrics					
	Optimizer	LR	Acc (%)	Sp (%)	Sn (%)	Pre (%)	F1-S (%)	AUC (%)
Augmented	SGD	0.0001	97.92	96.39	94.09	94.87	98.58	98.06
Augmented	ADAM	–	98.40	90.98	97.56	98.78	99.01	98.79

####  Results of the attention-GRU (A-GRU) on original data set

**Figure 12:**
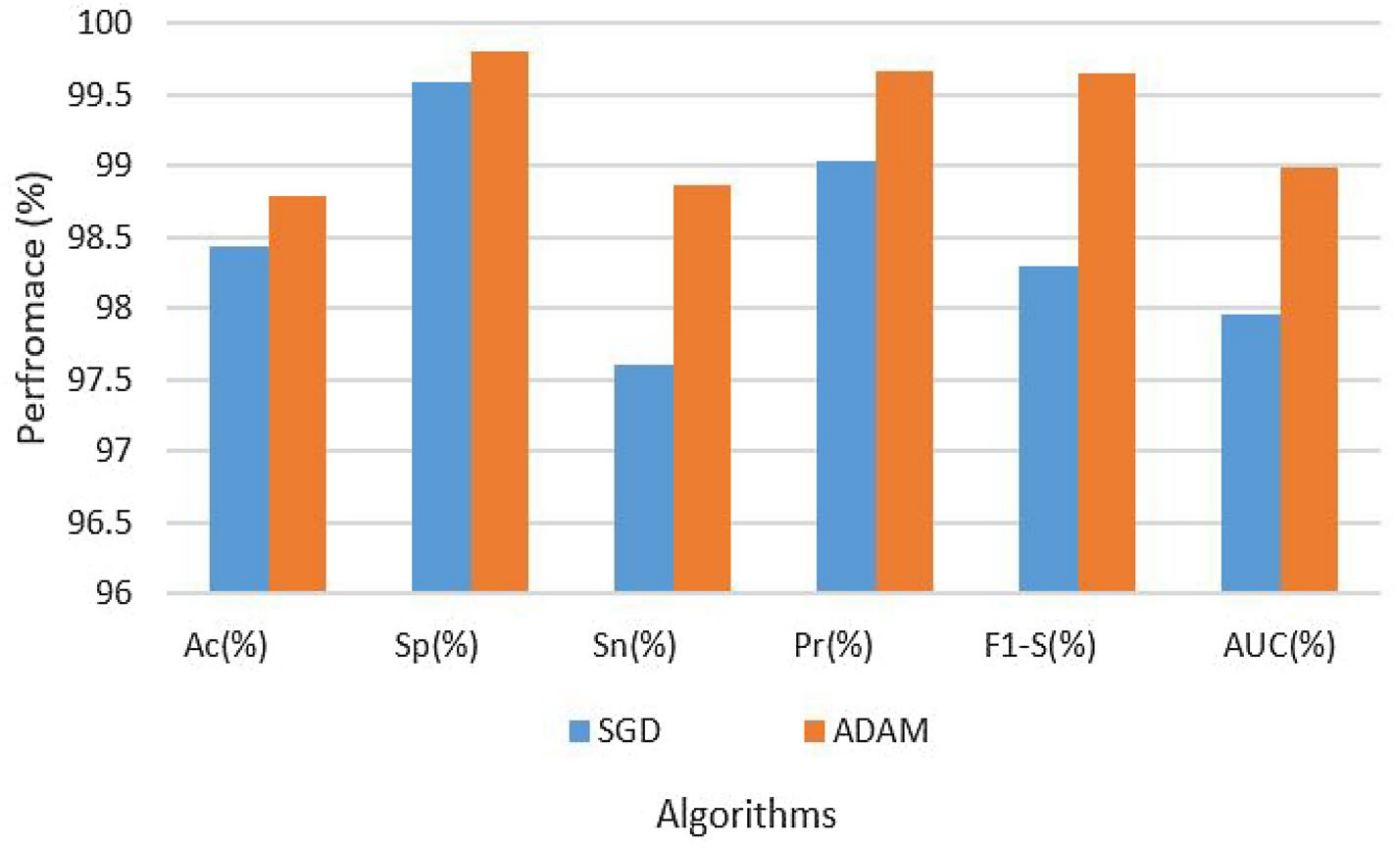
Results of A-GRU on original dataset.

A detailed summary of the hyperparameters and experimental outcomes is given in Table 10. The table highlights the attention-GRU model’s performance on the original dataset, utilizing two distinct optimization algorithms: SGD and ADAM, both configured with a fixed LR of 0.0001. It’s worth noting that the ADAM optimizer consistently outperforms SGD across the majority of evaluation metrics. When the model was used with the ADAM optimizer, it produced impressive results: 98.79% accuracy, 99.80% specificity, 98.87% robust sensitivity, 99.66% precision, 99.65% F1-Score, and 98.99% AUC. However, with an accuracy of 98.44%, specificity of 99.59%, sensitivity of 97.60%, precision of 99.03%, F1-Score of 98.30%, and AUC of 97.95%, the SGD optimizer yielded somewhat inferior results. These findings underscore the superior performance of the ADAM optimizer in delivering better overall model outcomes. These outcomes are also visually depicted in Fig. 12.

**Table 10:** Results of A-GRU on original dataset.

Data set	Parameters		Assessment metrics					
	Optimizer	LR	Acc (%)	Sp (%)	Sn (%)	Pre (%)	F1-S (%)	AUC (%)
Original	SGD	0.0001	98.44	99.59	97.60	99.03	98.30	97.95
Original	ADAM	–	98.79	99.80	98.87	99.66	99.65	98.99

####  Results of the proposed attention-GRU (A-GRU) on augmented data set

**Figure 13:**
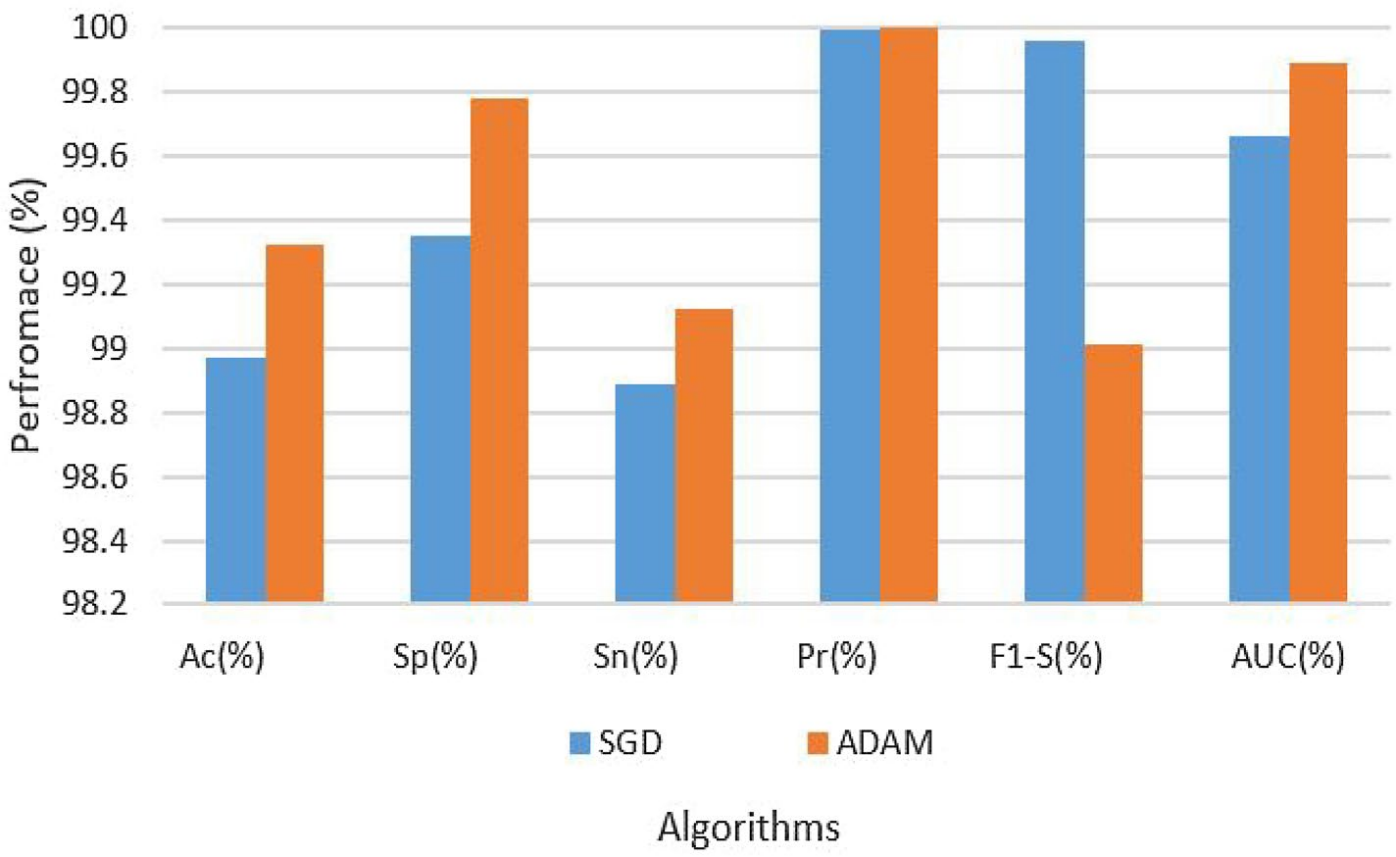
Results of proposed A-GRU on augmented dataset.

The performance of the attention-GRU model, as proposed, was assessed using augmented datasets. The model was configured with key hyperparameters and experimental results. Table 11 presents the findings, demonstrating that the proposed attention-GRU architecture, when applied to the augmented dataset using the SGD optimization algorithm with a learning rate (LR) of 0.001, delivered impressive performance metrics. Specifically, it achieved an accuracy of 98.97%, a specificity of 99.35%, a sensitivity of 98.89%, a precision of 99.99%, an F1-score of 99.96%, and an area under the curve (AUC) of 99.66%. Furthermore, when the proposed attention-GRU architecture was employed on the augmented dataset with the SGD optimization algorithm and the same LR of 0.001, it yielded notable results. The findings showed that when trained on the enhanced BTD, the accuracy was 99.32%, the specificity was 99.78%, the sensitivity was 99.12%, the precision was 100.00%, the F1-score was 99.01%, and the area under the curve (AUC) was 99.89%. The findings of these experiments led to the conclusion that the attention-GRU approach demonstrated remarkably high performance across all the augmented datasets. According to the information presented in Tables 2, 3, 4, 5, 6, 7, 8, 9, 10 and Table 11, our proposed model obtained remarkable results by using ADAM optimizer. When trained on the augmented BTD, the attention-GRU model’s accuracy rose to 99.32%. This underscores the significance of data augmentation when dealing with small original datasets. The outstanding performance of the proposed model is likely attributed to the careful tuning of hyperparameters and the implementation of data augmentation techniques. Moreover, the enhancements in performance extended to other metrics, including the specificity, sensitivity, precision, F1 score and AUC are 99.78%, 99.12%, 100%, 99.01% and 99.89% respectively. The experimental results lead us to the conclusion that the performance of the proposed attention-GRU with attention mechanism exhibited improvement across all evaluation metrics when the model was trained using the brain tumor dataset, regardless of whether the optimization algorithms employed are SGD and ADAM. The findings are also depicted in Fig. 13.

**Table 11:** Results of proposed A-GRU on augmented dataset.

Data set	Parameters		Assessment Metrics					
	Optimizer	LR	Acc (%)	Sp (%)	Sn (%)	Pre (%)	F1-S (%)	AUC (%)
Augmented	SGD	0.0001	98.97	99.35	98.89	99.99	99.96	99.66
Augmented	ADAM	–	99.32	99.78	99.12	100	99.01	99.89

####  Performance comparison of models on original and augmented data (CNN, A-CNN, LSTM, A-LSTM, GRU, A-GRU)

A comparison has been made between the performance of the several models (CNN, A-CNN, LSTM, A-LSTM, GRU, and A-GRU) in Table 12. The proposed model, attention-GRU with augmented data set, using ADAM optimizer achieved the highest accuracy at 99.32%, indicating its superior capability in making correct classifications. It outperforms all other models, including those with SGD and ADAM variations. Among models using ADAM optimizer, also exhibits the highest specificity at 99.78%, indicating its exceptional ability to correctly identify negative cases. Our proposed ADAM optimizer achieves a sensitivity of 99.12%, which is second only to the attention-GRU model with ADAM at 98.87%. This demonstrates its robustness in capturing positive cases. The proposed model using ADAM optimizer achieves a perfect precision of 100.00%, indicating that when it predicts a positive case, it is almost always correct. It significantly outperforms all other models. Our model also has one of the highest F1 scores at 99.01%, highlighting its ability to maintain a balance between precision and recall. Other models come close but do not surpass this score. Once again, our proposed model using ADAM optimizer leads the way with the highest AUC at 99.89%. This metric reflects its ability to discriminate between positive and negative cases, making it a strong performer in this regard. In conclusion, the proposed model, attention-GRU with augmented dataset consistently stands out as one of the top-performing models in terms of accuracy, precision, AUC, and overall balance between precision and recall. It demonstrates exceptional capabilities for classification tasks and offers a compelling benchmark for further research. When choosing a model for applications where accuracy and precision are crucial, the attention-GRU model with an augmented dataset using the ADAM algorithm is a strong candidate. Additionally, these models’ space and time complexity (CNN, A-CNN, LSTM, A-LSTM, GRU, A-GRU) are also reported in Table 12.

**Table 12:** Performance comparison of models on original and augmented data (CNN, A-CNN, LSTM, A-LSTM, GRU, A-GRU).

Model	Data set	Parameters		Complexity	Assessment Metrics						
		Optimizer	LR	SC	TC	Acc (%)	Sp (%)	Sn (%)	Pre (%)	F1-S (%)	AUC (%)
CNN	Original	SGD	0.0001	28.03M	5.05h	92.33	89.02	90.08	96.34	98.30	96.05
		ADAM	0.0001	28.01M	5.03h	92.84	93.30	95.34	97.00	97.89	97.93
CNN	Augmented	–	–	27.06M	4.07h	93.87	98.03	95.90	98.64	97.00	98.34
		-	–	27.07M	4.09h	93.98	99.10	97.34	98.89	98.33	98.90
A-CNN	Augmented	–	–	28.06M	5.02h	94.00	98.03	95.90	98.64	97.00	98.64
		–	–	28.08M	4.04h	95.78	98.77	98.94	99.09	99.34	99.00
LSTM	Original	–	–	39.02M	4.01h	96.30	90.50	92.28	98.98	98.00	98.84
		–	–	39.05M	9.08h	97.54	93.00	95.49	99.00	98.08	99.00
LSTM	Augmented	-	–	38.03M	5.08h	97.44	94.60	96.88	97.58	98.90	90.04
		–	–	39.02M	5.03h	97.54	95.77	97.01	98.66	98.98	99.70
A-LSTM	Augmented	–	–	37.01M	5.03h	98.00	97.82	97.18	87.00	99.30	99.04
		-	–	41.01M	5.04h	98.64	97.47	98.91	98.96	98.98	98.90
GRU	Original	–	–	110.01M	5.04h	97.90	98.09	99.00	98.34	99.00	98.97
		–	–	110.05M	5.04h	98.02	99.00	99.17	99.03	99.69	98.99
GRU	Augmented	–	–	113.05M	6.05h	97.92	96.39	94.09	94.87	98.58	98.06
		-	–	115.08M	6.04h	98.40	90.98	97.56	98.78	99.01	98.79
A-GRU	Original	–	–	121.07M	6.06h	98.44	99.59	97.60	99.03	98.30	97.95
		-	–	122.06M	7.09h	98.79	99.80	98.87	99.66	99.65	98.99
A-GRU	Augmented	–	–	122.03M	7.03h	98.97	99.35	98.89	99.99	99.96	99.66
		–	–	122.07M	7.08h	**99.32**	99.78	99.12	100	99.01	99.89

The number of trainable parameters in a model represents its space complexity. M stands for million. The more trainable parameters there are, the higher the space complexity (SC). The models’ training time (measured in hours) is known as the time complexity (TC).

Significant values are in [bold].

#### Comparison with baseline models

The performance of the proposed model A-GRU has been compared with baseline models in terms of accuracy and reported in 13. Table 13 presented that the proposed model obtained a high accuracy of 99.32% as compared to baseline models. The higher shows that the model is more suitable for brain tumor detection and can easily incorporated in the E-health care system.

**Table 13:** Comparison of A-GRU model accuracy with previous models.

Ref	Model	Acc (%)	Space Complexity	Time Complexity
^38^	NeuroNet19	99.3	$$\mathcal {O}(cwh + 1)f$$	$$\mathcal {O}(f*u*m)$$
^39^	HHOCNN	98%	$$\mathcal {O}(cwh + 1)f$$	$$\mathcal {O}(f*u*m)$$
^4^	CNN-LSTM	99.22	$\mathcal {O}((nm + mk) + (n*d) )$	$\mathcal {O}((cwh) + (nd+Kn))$
^11^	CNN and VGG16	93.36, 97.16	$$\mathcal {O}(cwh + 1)f$$	$$\mathcal {O}(f*u*m)$$
^14^	AE-DNN	96	$\mathcal {O}((n) + (n*d))$	$\mathcal {O}((n^2) + (nd+Kn))$
^15^	ACNN	96.7	$$\mathcal {O}(cwh + 1)f$$	$$\mathcal {O}(f*u*m)$$
2024	Proposed model A-GRU	99.32	$$\mathcal {O}(cwh + 1)f$$	$$\mathcal {O}(f*u*m)$$

$$c=$$ the number of convolutional channels, $$h=$$height of input, $$w=$$ width of input, $$f=$$ the convolutional kernel size, $$n=$$ the number data instances, $$k=$$ the number of output neurons, $$m=$$ the number of input neurons and $$d =$$ the dimension or feature of the input, $$K=$$number of nearest neighbors, $$u=c*w*h$$

#### Space and time complexity

The space and time complexity of various proposed models(CNN, A-CNN, LSTM, A-LSTM, GRU, A-GRU) with original and augmented data are reported in Table 12 for the detection of brain tumors. Since the proposed models are deep learning techniques, the trainable parameters of each model are taken into consideration when analyzing the space complexity. We utilize the model’s training time to calculate the temporal complexity. According to Table 12 A-GRU has the worst space complexity since its trainable parameter is 122.07 million, while LSTM has 4.01 h the best space-time complexity. Additionally, for the time complexity, the A-GRU model has the worst time complexity because its training time is 7.09 h. We were unable to empirically analyse the model complexity in terms of algorithmic run-time due to the challenges associated with gaining access to the models of the competing approaches listed in Table 13. Due to the large amount of parameters and matrix processing that come with the model’s architecture, it is more likely that nearly all methods using deep learning techniques-convolutional neural networks-will have a worse space and time complexity. Compared to all other competing methods, our suggested model provides an accuracy performance boost, even for the worst-case time and space complexity. The models’ training time (measured in hours) as shown in Table 13 represents the time complexity. Our model A-GRU has a space complexity of $$\mathcal {O}(cwh + 1)f$$ and a time complexity of $$\mathcal {O}(f*u*m)$$.

#### Discussion

The brain tumor’s accurate and on-time detection is necessary for proper treatment and recovery. Artificial intelligent (AI) Based CAD systems can effectively detect brain tumors. In this regard, various research proposed different brain tumor detection techniques using AI techniques, particularly machine learning and deep learning using clinical data. According to a literature review in “Literature review”, these techniques still have the problem of lack of diagnosis accuracy. To tackle the accurate diagnosis problem of brain tumors a more advanced method is necessary. This work presented a novel method that is based on deep learning techniques for the diagnosis of brain tumors. In the proposed method deep learning model GRU is used with attention techniques to accurately detect brain tumors. Different optimization algorithms SGD and ADAM were incorporated for effective training of the model. The proposed model tested on brain tumors MRI images data set using hold out validation technique. The data augmentation techniques were incorporated to enhance the data set for the effective training of the model. The model performances were evaluated using different evaluation metrics. The experimental results demonstrated that the proposed model A-GRU With the augmented dataset, accuracy improved from 98.79 to 99.32% throughout training. The proposed model has high accuracy due to the adjustment of hyperparameters and data augmentation techniques as compared to baseline models.

In the medical field, the attention-GRU approach is appropriate for diagnosing brain tumors, as it can be applied to a wide range of medical technologies. However, as the number of epochs increased and the learning rate decreased, the training time and memory consumption of the model increased. This is due to the increased computational complexity of the model. The study suggests that the attention-GRU method is suitable for brain tumor diagnosis in the medical healthcare systems. However, memory use was less when training the model without data augmentation than when training with augmented data.

## Conclusion

This research work explores the application of the attention-GRU model (A-GRU) for deep feature extraction and classification of brain tumors in magnetic resonance imaging (MRI) data. The model demonstrates its efficacy in handling the complex nature of brain tumor imaging, improving tumor classification accuracy and diagnosis reliability. The study compares CNN, LSTM, and the attention-GRU model, revealing their strengths and weaknesses in brain tumor classification. The attention-GRU model (A-GRU) combines the advantages of both CNN and LSTM by incorporating an attention mechanism, demonstrating superior performance in feature extraction and classification. The study also compares optimization algorithms, SGD and ADAM, and their impact on training deep learning models. The attention-GRU model has shown a remarkable capacity for learning and recognizing spatial and temporal features within MRI sequences, which is essential for the accurate classification of brain tumors. The proposed model A-GRU obtained 99.32% accuracy with augmented data as compared to baseline models. The high performance of the A-GRU model is due to the attention approach. However, model interpretability remains a significant concern, and ethical considerations such as patient privacy and data security should be paramount when implementing deep learning in a clinical setting. Thorough validation and extensive clinical trials are necessary to guarantee the model’s safety and effectiveness before its broad implementation. The study makes a substantial contribution to medical imaging and deep learning, illustrating the model’s potential to enhance patient outcomes and advance knowledge of brain tumors. In the future, we will incorporate Transfer learning and Federated learning techniques with different brain Tumor MRI datasets to further improve the predictive capability and design a state-of-the-art system for brain tumor detection.

## Data Availability

In this work, we used the Brian MRI dataset for our experiments and Brain MRI Scans for the brain tumor classification data sets are available on Kaggle repository https://www.kaggle.com/datasets/shreyag1103/brain-mri-scans-for-brain-tumor-classification.
